# Study on the Transverse Vibration Characteristics of Phenine Nanotubes

**DOI:** 10.3390/nano15040300

**Published:** 2025-02-16

**Authors:** Zhuoqun Zheng, Han Li, Lifeng Wang, Xu Xu, Eric Li

**Affiliations:** 1 State Key Laboratory of Mechanics and Control for Aerospace Structures, Nanjing University of Aeronautics and Astronautics, Nanjing 210016, China; 2 AVIC Xinxiang Aviation Industry (Group) Co., Ltd., Xinxiang 453049, China; 3 College of Mathematics, Jilin University, Changchun 130012, China; 4 Key Laboratory of Symbolic Computation and Knowledge Engineering of Ministry of Education, Jilin University, Changchun 130012, China; 5 School of Computing, Engineering & Digital Technologies, Teesside University, Middlesbrough TS1 3BX, UK

**Keywords:** phenine nanotubes, holes, geometrically equivalent beam model, molecular dynamics simulation, natural frequency

## Abstract

Phenine nanotubes are tubular molecular structures with periodic hexatomic vacancies. The holes formed by these vacancies have a significant impact on their electrical, mechanical, and other properties. In this paper, the transverse vibration characteristics of phenine nanotubes (PNTs) are investigated by molecular dynamics (MD) simulation and continuum mechanics. A geometrically equivalent beam model is established for describing the geometric characteristics of holes. The effective static mechanical parameters of PNTs used in the proposed model are calibrated by MD simulations. The first four-order natural frequencies of PNTs are predicted by MD simulations and geometrically equivalent beam models. The results indicate that the geometrically equivalent beam model performs well in describing the transverse vibration characteristics of PNTs. Furthermore, the applicability ranges of geometrically equivalent beam models are discussed. This study offers valuable insights into the transverse vibration characteristics of porous nanostructure, which would be beneficial for the design of nanoscale mechanical resonators.

## 1. Introduction

Since 2019, the successful synthesis of phenine nanotubes (PNTs) [1] attracted wide attention [2,3,4,5]. The large aspect ratio and porous structure of PNTs make them promising for potential applications in areas such as the desalination of seawater [6,7], gas separation [8], anode materials in Li-ion batteries [9], and nanomechanical resonators [10,11,12,13,14,15,16]. PNTs can be considered carbon nanotubes (CNTs) with periodic and regular pores on their walls. Compared with traditional CNTs, the structure of PNTs is more flexible, allowing for doping with other atoms or functional groups, such as hydrogen or nitrogen, at the pore site [4,17]. Moreover, the existence of pores makes it easier to adsorb small particles, which is beneficial for mass sensors. Hence, PNTs have potential applications in the field of micro–nano electromechanical systems. The arrangement of periodically regular pores does not affect the structural stability of PNTs. Furthermore, a study based on density functional theory has demonstrated that (*n*,*n*) PNTs exhibit favorable electronic properties, making them wide-bandgap semiconductor materials [3]. Compared with CNTs, the hydrogenation at the pores of PNTs broadens their range of absorption and emission of light, potentially enhancing their luminescent capabilities [18]. The excellent optical properties of PNTs also demonstrate the potential of structural void engineering and surface functionalization in obtaining multifunctional tubular materials. The mechanical properties of PNTs based on molecular dynamics (MD) simulations have been investigated. Yu and Yang [19] conducted a static analysis of (*n*,*n*) PNTs using MD methods. It is found that the relaxed stable structure of the cross-section of PNTs exhibits a polygonal shape due to the twisting of continuous C-C chains. Furthermore, it is observed that as the diameter increases, the number of edges in the cross-section of the structure also increases. However, this trend does not continue indefinitely. When the diameter reaches 2.48 nm, the cross-section of the structure collapses and no longer maintains a polygonal shape [20]. The presence of pores inevitably results in the mechanical strength of PNTs being weaker than that of non-porous CNTs. While this is predictable, it is still necessary to systematically explore their static mechanical properties. Faria and Silvestre [21] employed MD methods to elucidate the differences in static mechanical parameters between PNTs and CNTs. Both PNTs and CNTs were equivalently modeled as hollow cylinders in the framework of continuum mechanics. The stretching, compression, torsion, and bending tests for chiral and zigzag PNTs under the AIREBO potential [22] and Reax potential [23] were conducted. The critical stresses under different loading conditions, as well as mechanical parameters such as elastic modulus and shear modulus, were calculated. In this study, the influence of pores was not taken into account when calculating the static mechanical parameters.

The aforementioned work has conducted preliminary studies on the physical and static mechanical properties of PNTs. However, currently, there is no reported research on the dynamic characteristics of PNTs. The classical beam models, including the Euler–Bernoulli model and the Timoshenko model, have been demonstrated to accurately describe the lateral vibration characteristics of CNTs [24,25,26,27,28]. Given the structural differences between PNTs and CNTs, a geometrically equivalent beam model considering the presence of pores [29,30] can be introduced into the classical beam models for the study of lateral vibration characteristics in PNTs. The establishment of a geometrically equivalent beam model involves the calculation of various equivalent geometric parameters and equivalent static mechanical parameters. While the calculation methods for these equivalent parameters have been extensively studied in macroscopic porous structures [31,32,33], they may not be directly applicable to the calculation of equivalent static mechanical parameters for PNTs. Molecular dynamics simulation, as a commonly used method to describe the mechanical behavior of nanostructures, is beneficial for addressing this issue. The accurate calibration of equivalent static mechanical parameters for PNTs is crucial to ensuring the effective description of their lateral vibration characteristics using the geometrically equivalent beam model [29]. 

This paper focuses on the transverse vibration characteristics of PNTs. The natural frequencies of PNTs under clamped–clamped boundary conditions were investigated by MD simulations and geometrically equivalent beam models. Geometrical parameters were calibrated based on the structure analysis of PNTs. Then, MD simulations were conducted to perform tensile, torsional, and bending tests of the model for the calibration of its static mechanical parameters. By comparing the natural frequencies of MD simulations and proposed beam models, it is found that the geometrically equivalent Timoshenko beam model can effectively predict the natural frequencies of PNTs. Furthermore, the shear coefficient *k* was also discussed. This paper provides a set of methods dealing with the prediction of dynamic properties of tubular structures with regular pores.

## 2. Molecular Dynamics Simulation

**Figure 1:**
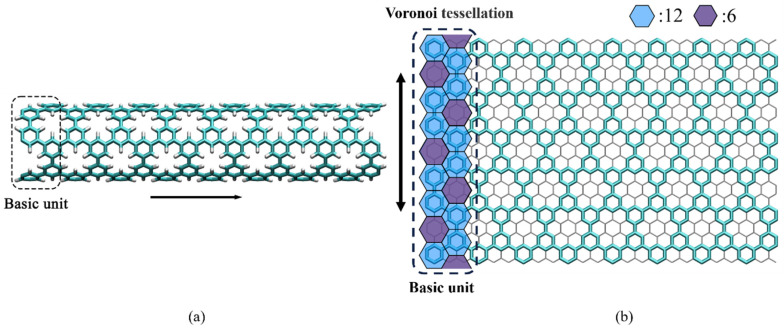
Atomic configuration of the PNTs: (**a**) Atomic structure diagram; (**b**) Unfolded structural diagram.

PNTs are tubular molecular structures synthesized from benzene rings with substitutions 1, 3, and 5 [1]. As shown in Figure 1a, from a structural perspective, it can be approximately regarded as CNTs with periodic pores, where unsaturated carbon atoms at pore sites are passivated by hydrogen atoms. The atomic models of PNTs with different lengths can be achieved by periodically replicating varying numbers of basic units. To better observe the structural characteristics of PNTs, the structure was illustrated in an unfolded schematic diagram, as shown in Figure 1b. A basic unit was a token as the analysis object. Perpendicular bisectors between two adjacent atoms were drawn, and several Voronoi polygons were formed by the intersections of these perpendicular bisectors as seen in Figure 1b. The blue hexagons represent regions occupied by carbon atoms, while the purple hexagons represent the areas occupied by pores. Within a single unit, there are 12 blue hexagons and 6 purple hexagons, indicating a ratio of 2:1 between the regions occupied by carbon atoms and those occupied by pores. This provides a crucial theoretical basis for subsequent calculations of its mechanical parameters and geometric cross-sectional parameters. In the previous synthetic study [1], the (9,9), (12,12), and (15,15) PNTs are reported. Due to the limitation of computing resources, (9,9) PNTs are mainly investigated in this study. Furthermore, (12,12) PNTs are utilized to validate the proposed calibration methods and beam models. The diameters of (9,9) and (12,12) PNTs are 1.22 nm and 1.63 nm, respectively.

All MD simulations in this study were carried out using the open-source Large-scale Atomic/Molecular Massively Parallel Simulator (LAMMPS) code [34], and the Reax potential proposed by Chenoweth et al. [23] was employed to describe the atomic interactions of PNTs. This force field effectively captures the interactions between C-C bonds, C-H bonds, and non-bonded interactions, allowing for accurate simulation of mechanical processes such as necking, buckling, and twisting, albeit with the drawback of higher computational costs.

The relaxation of the model was performed under periodic boundary conditions, which effectively released axial stress. To ensure the elimination of axial stress after relaxation, the initial axial stress of the model was calculated. When the absolute value of axial stress was less than 0.02, it means that the axial stress of the model had been eliminated. At this point, the structure is in an optimal position with the lowest energy state, allowing for subsequent mechanical simulations.

**Figure 2:**
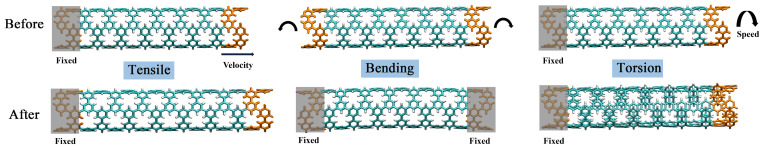
MD simulation settings of the tensile, bending, and torsional tests of PNTs.

The tensile and torsional tests were all conducted under non-periodic boundary conditions and the NVT ensemble with a temperature of 10 K. The time step for all these processes was 0.25 fs. The length of the relaxed model was chosen as 38.86 nm. For the tensile tests [28], one end of the relaxed model was fixed and then tensile load (a constant axial velocity) was applied to the other end as shown in Figure 2. The tensile strain rate was set to 10^−7^/fs. For the torsional tests [28], one end of the relaxed model was fixed and then torsional load (a constant rotational velocity) was applied to the other end as shown in Figure 2. The torsional rate was set to 6 × 10^−7^ rad/fs.

The bending tests were also conducted under non-periodic boundary conditions [35,36]. Firstly, the relaxed model was set along *z* axis, and the center of cross-section was moved to (0,1/*c*,*z*), where *c* is the targeted curvature. Then, the relaxed model can be bent to the targeted curvature through the following coordinate transformation:

(1) $$\left\{\begin{array}{l} x_{\mathrm{Curved}}=x,\\ y_{\mathrm{Curved}}=y\cos(cz),\\ z_{\mathrm{Curved}}=y\sin(cz). \end{array}\right.$$

Subsequently, two ends of the model are fixed, and energy minimization is carried out. After the energy minimization, the stable potential energy of the model at that curvature is output. The energy minimization in the bending simulation is performed using the Fast Inertial Relaxation Engine (FIRE) method.

For the thermal vibration tests, both ends of the relaxed model were fixed to mimic the clamped–clamped boundary conditions. Then, the other parts of the relaxed model vibrate freely under the NVT ensemble.

## 3. Geometrically Equivalent Beam Model

There are many beam theories [25,37,38,39] that can describe the transverse vibration characteristics of beams. Among them, the Euler–Bernoulli and Timoshenko beam theories are the most widely used theories for slender beams [39]. Since PNTs are porous structures as shown in Figure 1, the effect of pores should be considered in the beam models. Due to the regular distribution of pores in PNTs, the effect of holes can be described through equivalent geometrical parameters. Hence, the expression for the geometrically equivalent Timoshenko beam is [28,30,39].

(2) $$E_{s}I_{eq}\frac{\partial^{4}u}{\partial x^{4}}+\rho_{s}A_{eq}\frac{\partial^{2}u}{\partial t^{2}}-\left(\rho_{s}I_{eq}+\frac{E_{s}I_{eq}\cdot \rho_{s}A_{eq}}{kAG_{eq}}\right)\frac{\partial^{4}u}{\partial x^{2}\partial t^{2}}+\frac{\rho_{s}I_{eq}\cdot \rho_{s}A_{eq}}{kAG_{eq}}\frac{\partial^{4}u}{\partial t^{4}}=0.$$

Here, $u=u\left(x,t\right)$ is the lateral coordinate of the deflection axis of the porous beam. In the equation, $E_{s}I_{eq}$ represents the flexural rigidity, which is the product of the elastic modulus $E_{s}$ and the equivalent moment of inertia $I_{eq}$ of the porous beam’s cross-section. The equivalent unit length mass $\rho_{s}A_{eq}$ is the product of the material density $\rho_{s}$ and the equivalent cross-sectional area $A_{eq}$ of the porous beam. The equivalent shear stiffness $AG_{eq}$ includes the structural shear modulus $G_{eq}$ of the porous beam. *k* is the Timoshenko shear coefficient, which depends on the geometry. The last term $\rho_{s}I_{eq}$ is the equivalent rotational inertia of the unit-length beam about the cross-sectional inertia principal axis.

In practical applications, Equation (2) can be simplified. Previous studies [39] indicate that, based on classical beam theory, the term equivalent rotational inertia $\rho_{s}I_{eq}$ is significantly smaller than the shear deformation term $E_{s}I_{eq}\cdot \rho_{s}A_{eq}/AG_{eq}$ for common materials and cross-sections. Therefore, the influence of rotational inertia is often neglected. Additionally, when their shear stiffness is sufficiently large, shear deformation can be neglected in low-order vibration modes of slender beams. If both the rotational inertia and shear stiffness are ignored, Equation (2) can be degenerated into the geometrically equivalent Euler–Bernoulli beam model [28,30,39].

(3) $$E_{s}I_{eq}\frac{\partial^{4}u}{\partial x^{4}}+\rho_{s}A_{eq}\frac{\partial^{2}u}{\partial t^{2}}=0.$$

The solution of this form is typically assumed as

(4) $$u\left(x,t\right)=U\left(x\right)\cos\left(\omega_{n}t\right).$$

$\omega_{n}$ is the (*n*)-th order circular natural frequency, the coefficient n=1,2,3…. Substituting Equation (4) into Equation (2), we can obtain an ordinary differential equation

(5) $$E_{s}I_{eq}\frac{d^{4}U}{dx^{4}}-\rho_{s}A_{eq}{\omega_{n}}^{2}U+\left(\rho_{s}I_{eq}+\frac{E_{s}I_{eq}\cdot \rho_{s}A_{eq}}{kAG_{eq}}\right){\omega_{n}}^{2}\frac{d^{2}U}{dx^{2}}+{\omega_{n}}^{4}\frac{\rho_{s}A_{eq}\cdot \rho_{s}{Ι}_{eq}}{kAG_{eq}}U=0.$$

Substituting Equation (4) into Equation (3), we can obtain an ordinary differential equation

(6) $$E_{s}I_{eq}\frac{d^{4}U}{dx^{4}}-\rho_{s}A_{eq}{\omega_{n}}^{2}U=0.$$

By applying the clamped–clamped boundary conditions [28], characteristic equations can be obtained. Then, natural frequencies are determined by numerically solving [40].

## 4. Results and Discussion

### 4.1. Calibration of Geometrical Parameters for PNTs

**Figure 3:**
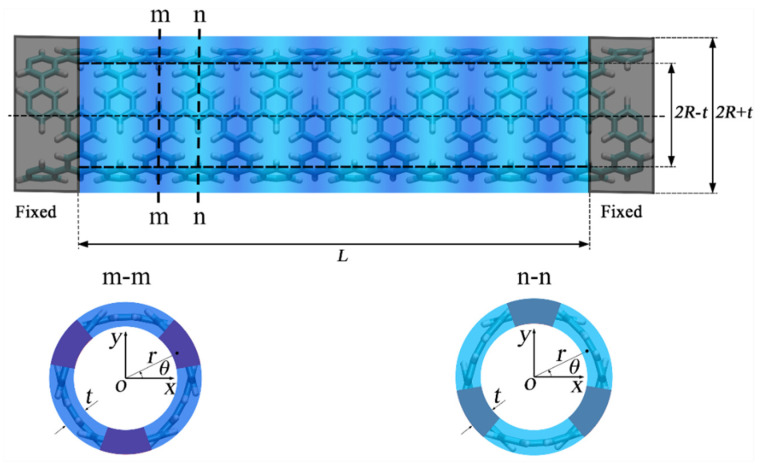
PNTs under clamped–clamped boundary conditions and cross-sectional views at different positions.

Based on the principles of continuum mechanics, PNTs can be equivalently modeled as hollow cylinders with radius *R* and thickness *t*, as illustrated in Figure 3. The gray region represents the fixed boundaries set during MD simulations, and the effective length *L* in theoretical calculations is the blue region. The blue region is composed of alternating deep and light blue sections, indicating two predominant cross-sectional forms of PNTs, referred to as the m-m cross-section and n-n cross-section, as shown in Figure 3. The purple region in the cross-section represents the porous area, and the arrangement of the cross-section is periodic, similar to the periodicity of the pores. We calculate the equivalent sectional moments of inertia and areas for PNTs based on these two cross-sectional forms.

For the sector-shaped cross-section with notches, the expression for calculating the sectional moment of inertia with respect to the principal axis *x* in polar coordinates is

(7) $$I_{xx}={\int_{R_{1}}^{R_{2}}r^{3}dr\int_{\theta_{1}}^{\theta_{2}}\left(\sin\theta \right)}^{2}d\theta +{\int_{R_{1}}^{R_{2}}r^{3}dr\int_{\theta_{3}}^{\theta_{4}}\left(\sin\theta \right)}^{2}d\theta +{\int_{R_{1}}^{R_{2}}r^{3}dr\int_{\theta_{5}}^{\theta_{6}}\left(\sin\theta \right)}^{2}d\theta .$$

Among them, the inner diameter $R_{1}=R-t/2$, outer diameter $R_{2}=R+t/2$, and the ones with subscripts θ represent three solid regions. The angles of the solid regions can be determined based on the distribution and proportion of carbon atom regions and pore regions.

In the Timoshenko beam theory, the bending stiffness EI, which is the product of the sectional moment of inertia I and the elastic modulus E, has a significant impact on the transverse vibration frequency. From Figure 1a, it can be seen that the average flexural radius of the beam is the weighted average of the bending radii of the two cross-sectional segments, and the bending radius is inversely proportional to the bending stiffness. Therefore, the expression for the equivalent sectional moment of inertia $I_{eq}$ is

(8) $$\frac{1}{I_{eq}}=\frac{1}{2}\frac{1}{I_{xx\left(m-m\right)}}+\frac{1}{2}\frac{1}{I_{xx\left(n-n\right)}}.$$

It can be further simplified to

(9) $$I_{eq}=I_{xx\left(m-m\right)}=I_{xx\left(n-n\right)}=\frac{2}{3}I.$$

Similarly, the expression for the equivalent sectional area is

(10) $$A_{eq}=\frac{1}{2}A_{m-m}+\frac{1}{2}A_{n-n},$$

(11) $$A_{eq}=A_{m-m}=A_{n-n}=\frac{2}{3}A.$$

The equivalent thickness is calculated based on the tensile stiffness obtained from stretching simulations and the bending stiffness obtained from bending simulations. The relationship between the two is as follows:

(12) $$\frac{K}{EA}=\frac{D}{EI}.$$

From this formula, it can be deduced that the equivalent thickness of both PNTs and CNTs is 0.29 nm.

### 4.2. Calibration of Mechanical Parameters for PNTs

In the geometrically equivalent Timoshenko beam model proposed in the second section, four equivalent parameters are involved. These are the equivalent bending stiffness $E_{s}I_{eq}$, equivalent shear stiffness $AG_{eq}$, equivalent unit length mass $\rho_{s}A_{eq}$, and equivalent unit length moment of inertia $\rho_{s}I_{eq}$. Among them, the calculation of static mechanical parameters is achieved through MD simulations. The calibration of each static mechanical parameter is discussed in this section. In order to better understand the stress conditions of PNTs under different static simulations, a comparison with CNTs is made for each parameter.

**Figure 4:**
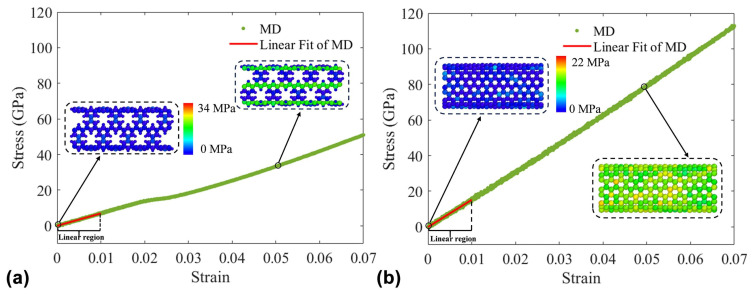
Stress–strain curves and atomic stress distribution. (**a**) (9,9) PNT with a length of 38.86 nm; (**b**) (9,9) CNT with a length of 39.14 nm.

Tensile simulations of PNTs are shown in Figure 4, where (a) and (b) represent the stress–strain curves and atomic stress distributions of (9,9) PNTs with a length of 38.86 nm and (9,9) CNTs with a length of 39.14 nm, respectively. From the slope, it can be observed that the elastic modulus of (9,9) PNTs is smaller than that of (9,9) CNTs. The calculation of the elastic modulus involves selecting the linear region data with axial strain less than 0.01 from Figure 4. Upon calculation, Young’s elastic moduli are 691.53 GPa and 1525.42 GPa, respectively. Compared with the well-known CNT elastic modulus of 1 TPa, the calculated value seems to be slightly larger. This is because the Reax potential and the equivalent thickness of 0.29 nm are utilized in our calculations. Furthermore, we have separately plotted the atomic stress distribution for both cases at axial strains of 0 and 0.05. The color scale, ranging from blue to red, represents the increasing atomic stress. Upon careful observation, it is noticed that the axial force acting on (9,9) PNTs is mainly concentrated on a few continuous carbon atomic chains, whereas the axial force on (9,9) CNTs is more evenly distributed among all carbon atoms. This phenomenon indicates that the presence of pores alters the number of carbon atoms bearing the axial force in the structure, which is a significant reason for its lower elastic moduli compared to CNTs.

Shear deformation effectively affects the higher-order transverse vibration frequencies of beams. For PNTs, the presence of pores significantly alters their shear modulus. When PNTs undergo shear, their deformation is no longer purely shear deformation but a combined deformation caused by bending and shear actions. Therefore, we use $G_{eq}$ to represent the equivalent shear modulus of PNTs under the combined action of bending and shear deformation and use $AG_{eq}$ to represent the equivalent shear stiffness.

The calculation of $G_{eq}$ here involves performing MD simulations for the torsional deformation of PNTs. The process involves recording the torque variation with end rotation during torsion simulations and calculating the equivalent shear modulus using the following relationship [28]:

(13) $$G_{eq}=\frac{ML}{\varphi {Ι}_{p}},$$

where ${Ι}_{p}$ is the polar moment of inertia of the entire beam, M is the torsional torque, φ is the end rotation angle, and L is the length of the model.

**Figure 5:**
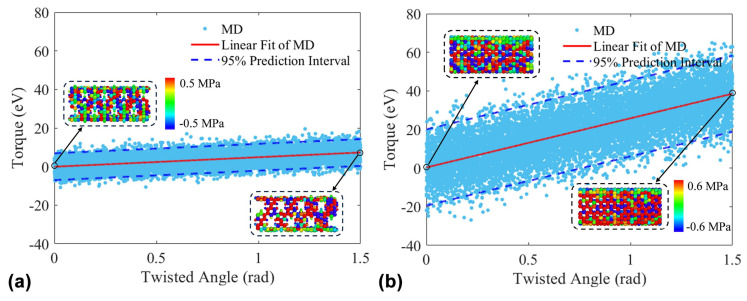
Scatter plot distribution of torque and atomic stress distribution. (**a**) (9,9) PNT with a length of 38.86 nm; (**b**) (9,9) CNT with a length of 39.14 nm.

During the torsion process, the torsional torque is recorded every 1.5 × 10^−4^ rad of end rotation. A scatter plot of the torsional torque experienced at the end is then plotted against the increasing rotation angle (0–1.5 rad), as shown in Figure 5. Figure 5a presents the torque scatter plot for the (9,9) PNT, and Figure 5b shows the torque scatter plot for the (9,9) CNT. The blue dots in the figures represent the torque data from MD simulations. Due to the thermal fluctuation, the torque data fluctuates over a large range. A similar phenomenon can be found in the previous study [28]. In order to obtain the reliable shear modulus, the torque data within the 95% confidence interval are used for linear fitting of torque and rotation angle, as shown by the red line in the figure. By comparing the slopes of the linear fits, it is evident that the shear modulus of the (9,9) PNT is significantly smaller than that of the (9,9) CNT. The shear modulus values for the (9,9) PNT and the (9,9) CNT are 70.06 GPa and 365.35 GPa, respectively. To further reveal the microscopic mechanism behind this phenomenon, the atomic stress distributions for each at 0 rad and 1.5 rad have been plotted, respectively. It can be visually observed that when subjected to shear stress, the number of atoms resisting shear deformation on the 45° shear plane of the (9,9) PNT is less than that of the (9,9) CNT. This also indicates that the presence of pores weakens the ability of the PNT to resist shear deformation.

**Figure 6:**
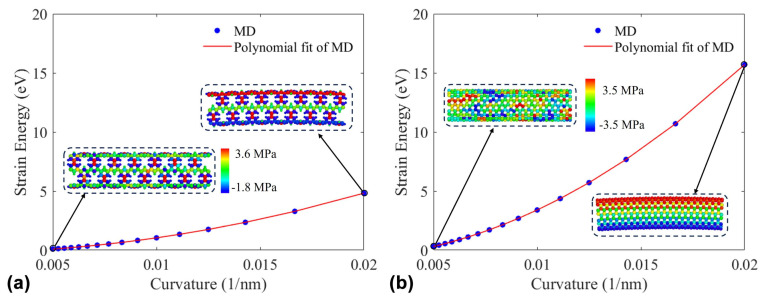
Bending strain energy with curvature and the distribution of atomic stresses. (**a**) (9,9) PNT with a length of 38.86 nm; (**b**) (9,9) CNT with a length of 39.14 nm.

Bending stiffness is a crucial mechanical parameter of materials. Here, MD simulations are employed to study the bending behavior of PNTs in order to determine their bending stiffness. Following the steps in Section 2, the stable potential energies of PNTs at different curvatures are obtained. The bending strain energy ΔE is calculated from $\Delta E=E_{b}-E_{0}$, where *E*_b_ is the potential energy of the current curved model and *E*_0_ is the potential energy of the model before bending. Figure 6 plots the bending strain energy as a function of the curvature. Figure 6a,b represent the variation in bending strain energy with curvature for (9,9) PNTs and (9,9) CNTs, respectively. In the figures, blue dots represent the bending strain energy at different curvatures obtained from MD simulations, while the red line represents a quadratic curve fitted to the MD data. The bending strain energy and curvature satisfy the following relationship [35,36].

(14) $$\Delta E=\frac{1}{2}DL\gamma^{2},$$

where ΔE represents the bending strain energy, D is the bending stiffness, γ is the curvature, and L is the length of the model. According to Equation (14), the bending stiffness for each can be calculated as $1.02\times 10^{-7}\;N\cdot nm^{2}$ and $3.33\times 10^{-7}\;N\cdot nm^{2}$.To further understand the differences in bending stiffness between the two, the atomic stress distribution is examined for both at the maximum curvature in Figure 6. From the color distribution, where blue represents compression and red represents tension, it is obvious that the distribution of blue and red layers is clear and roughly equal. Firstly, it indicates the validity of the implemented bending simulation. Secondly, it is evident that the presence of pores results in a lower number of atoms resisting bending deformation on the tube wall of (9,9) PNTs compared to (9,9) CNTs. This leads to a lower bending stiffness for (9,9) PNTs compared to (9,9) CNTs.

### 4.3. Transverse Vibration Characteristics of PNTs 

**Figure 7:**
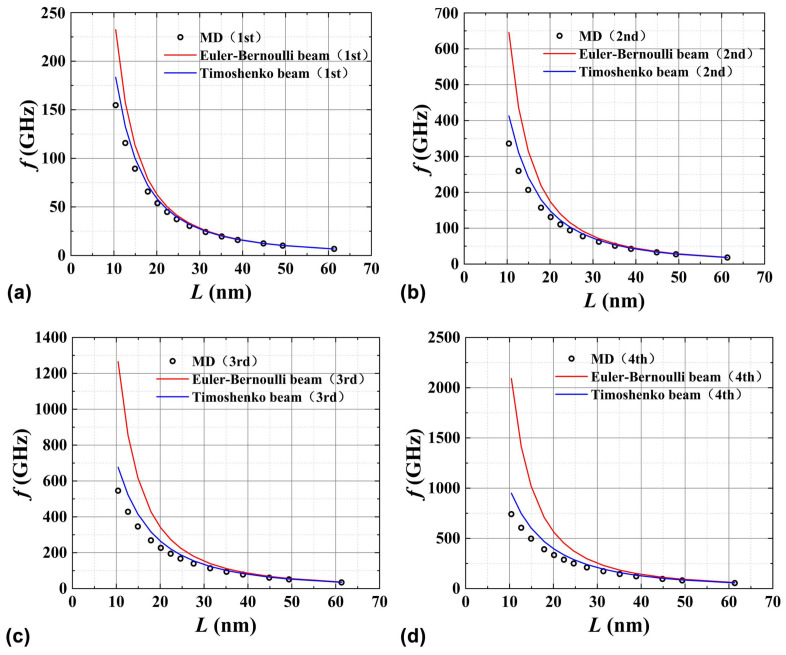
Geometrically equivalent Euler–Bernoulli beam, geometrically equivalent Timoshenko beam model, and MD simulations results of lateral vibration frequencies of (9,9) PNTs under fixed-support boundary conditions at both ends. (**a**) First-order frequency; (**b**) Second-order frequency; (**c**) Third-order frequency; (**d**) Fourth-order frequency.

Through the MD simulations of PNTs’ thermal vibrations, the temporal evolutions of the PNTs’ atoms can be obtained. Furthermore, by performing a fast Fourier transform (FFT) on the centroid coordinates of certain atoms, different-order natural frequencies of the lateral vibrations can be extracted at the same time. On the other hand, by incorporating the calculated mechanical and geometric parameters (summarized in Table 1) into the geometrically equivalent beam models, the natural frequencies of PNTs can be predicted. Figure 7 illustrates the natural frequencies of (9,9) PNTs under clamped–clamped boundary conditions for different vibration modes predicted by the geometrically equivalent Euler–Bernoulli beam model (red curve), Timoshenko beam model (blue curve), and MD simulations (black scatter points). Figure 7a–d, respectively, show the frequency variations with the length of PNTs predicted by the three methods for the first- to fourth-order vibration modes. From Figure 7, it is evident that the predictions of the geometrically equivalent Timoshenko beam model outperform those of the geometrically equivalent Euler–Bernoulli beam model. This observation highlights the effective correction of shear deformation in lateral vibration predictions. Furthermore, with increasing length, the predictions of both beam theories converge toward the results obtained from MD simulations.

To further illustrate the effectiveness of the geometrically equivalent beam models in describing the transverse vibration characteristics of PNTs, the transverse vibration frequencies of (9,9) and (12,12) PNTs with an aspect ratio of 32 are provided in Table 2. From the results, it can be observed that the natural frequencies predicted by the geometrically equivalent Timoshenko beam model are closer to those from MD simulations compared to the predictions of the geometrically equivalent Euler–Bernoulli beam model. Moreover, the maximum error of the fourth-order frequency predicted by the Timoshenko beam model does not exceed 10%. This highlights the capability of the geometrically equivalent Timoshenko beam model in accurately describing the transverse vibration characteristics of PNTs.

### 4.4. Applicability Range

**Figure 8:**
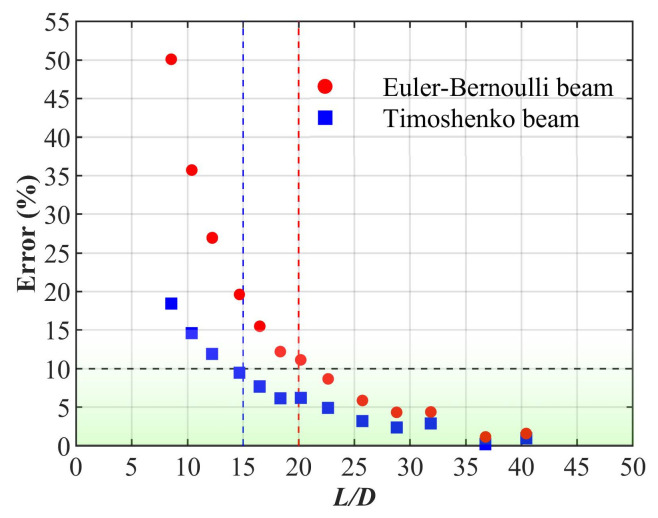
The errors between the first-order natural frequencies of (9,9) PNTs predicted by geometrically equivalent beam models and obtained from MD simulations with various aspect ratios under clamped–clamped boundary conditions.

This section focuses on the applicability ranges of the proposed geometrically equivalent models. Figure 8 provides the first-order frequency errors of the transverse vibration of (9,9) PNTs with different aspect ratios. The red points represent the errors between the geometrically equivalent Euler–Bernoulli beam models and MD simulations, while the black points represent the errors between the geometrically equivalent Timoshenko beam model and MD simulations. With the increase in the aspect ratio, the errors between the predictions by the geometrically equivalent beam models and the results from MD simulations gradually decrease. Moreover, it is evident that the predictions of the geometrically equivalent Timoshenko beam model are superior to those of the geometrically equivalent Euler–Bernoulli beam model. To discuss the applicability range of the two geometrically equivalent beam models in describing the transverse vibration characteristics of PNTs with both ends clamped, a criterion of a 10% error in the first-order vibration frequency is set as indicated by the black dashed line in the figure. In other words, when the error between the predictions of the two models and the MD simulation results exceeds 10%, it is considered that the geometrically equivalent beam models are not suitable for that aspect ratio. Thus, from Figure 8, it can be observed that for the geometrically equivalent Euler–Bernoulli beam model, it becomes inapplicable when its aspect ratio is less than 20. In contrast, for the geometrically equivalent Timoshenko beam model, this threshold decreases to 15. This indicates that the geometrically equivalent Timoshenko beam model, which considers shear deformation, expands the applicability range for describing the transverse vibration of PNTs compared to the geometrically equivalent Euler beam model. 

**Figure 9:**
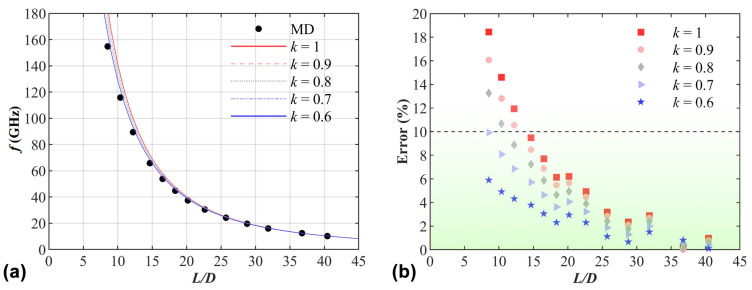
Under different values of *k* from 0.6 to 1, (**a**) shows the first-order natural frequencies of (9,9) PNTs predicted by geometrically equivalent beam models and obtained from MD simulations with various aspect ratios; (**b**) presents the errors between them.

On the other hand, compared to the applicability range of classical beam models for describing transverse vibrations in macroscopic structures, the aspect ratios of 20 and 15 as the thresholds for the geometrically equivalent Euler–Bernoulli beam model and the geometrically equivalent Timoshenko beam model, respectively, seem somewhat large. Then, we revisit the geometrically equivalent Timoshenko beam model. It is noticed that there is a shear coefficient *k* in Equation (2), which is aimed to compensate for the error caused by assuming a constant shear strain–stress through the thickness of the beam. In the above calculations, *k* is set to 1, which means that shear correction is not considered. Since the cross-sections of holey PNTs are quite complicated, it is necessary to take the shear correction into consideration. Although many researchers have discussed the calculation of the shear coefficient [41,42,43,44], it is still difficult to decide the value of the shear coefficient for PNTs due to the existence of holes in their structures. Hence, *k* values from 0.6 to 1 are tested. Figure 9a shows the first-order natural frequencies of (9,9) PNTs predicted by geometrically equivalent beam model and obtained from MD simulations under different values of *k*. It can be easily found that with the decrease in *k*, the frequencies predicted by the model gradually close to those from MD simulations. The errors between them are presented in Figure 9b. It can be easily found that when *k* drops to 0.7 or below, the threshold of the applicability range for the geometrically equivalent Timoshenko beam model decreases to 10 or below.

## 5. Conclusions

In this work, we proposed a method to calibrate the geometrical and static mechanical parameters of PNTs via MD simulations. The results indicate that the geometrically equivalent beam models based on pores can effectively describe the transverse vibration characteristics of PNTs. For predicting the natural frequencies, the geometrically equivalent Timoshenko beam model has a better performance than the geometrically equivalent Euler–Bernoulli beam model. It means that shear deformation plays a crucial role in the transverse vibration of PNTs. Furthermore, the applicable ranges of the geometrically equivalent beam models are investigated. The critical aspect ratio of the applicability range for the geometrically equivalent Timoshenko beam model reaches 10 when *k* is 0.7 or below. In conclusion, this paper systematically elaborated on the calibration method for the static parameters of PNTs and employed the geometrically equivalent beam models to describe the transverse vibration characteristics of PNTs. This study sheds light on the investigation of porous nanostructures, which has significant implications for the design and manufacturing of nanomechanical resonators.

## Figures and Tables

**Table 1:** Material properties of (9,9) PNT.

PNT	Diameter (nm)	Density (kg/m^3^)	Young’s Modulus (GPa)	Shear Modulus (GPa)
(9,9)	1.22	2690	696.86	70.06

**Table 2:** A comparison of lateral vibration frequencies of PNTs with different sizes predicted by the geometrically equivalent beam models and MD simulations.

PNT	*N*	MD	EB		TB	
		*f* (GHz)	*f* (GHz)	Error (%)	*f* (GHz)	Error (%)
(9,9)	1	16	16.7	4.38	16.46	2.88
	2	42.4	46.4	9.43	44.2	4.25
	3	78.6	90.94	15.7	83.85	6.68
	4	122.6	150.32	22.61	133.33	8.75
(12,12)	1	12.4	12.62	1.77	12.5	0.08
	2	32.8	35.04	6.83	33.73	2.84
	3	61.4	68.69	11.87	64.39	4.87
	4	96.6	113.54	17.54	103.09	6.72

## Data Availability

Data are contained within the article.
